# Clinical Recovery and Circulating Botulinum Toxin Type F in Adult Patient

**DOI:** 10.3201/eid1506.070571

**Published:** 2009-06

**Authors:** Jeremy Sobel, Tracy Dill, Christina L. Kirkpatrick, Laurel Riek, Patrick Luedtke, Todd A. Damrow

**Affiliations:** Centers for Disease Control and Prevention, Atlanta, Georgia, USA (J. Sobel); St. Peter’s Hospital, Helena, Montana, USA (T. Dill, C.L. Kirkpatrick); Lewis & Clark City-County Health Department, Helena (L. Riek); Utah Department of Health, Salt Lake City, Utah, USA (P. Luedtke); Montana Department of Health and Human Services, Helena (T.A. Damrow)

**Keywords:** Botulism, type F, botulinum toxin, antitoxin, flaccid paralysis, cost of illness, Montana, USA, dispatch

## Abstract

A 56-year-old woman in Helena, Montana, USA, who showed clinical signs of paralysis, received antitoxins to botulinum toxins A, B, and E within 24 hours; nevertheless, symptoms progressed to complete quadriplegia. On day 8, she began moving spontaneously, even though blood tests later showed botulinum toxin type F remained.

Botulism is a disease characterized by cranial nerve palsies and descending, symmetric, flaccid paralysis. Seven serologically distinct botulinum toxins, designated A through G, are known; virtually all human cases are caused by types A, B, E, and rarely, F (*1*). *Clostridium botulinum* produces all 7 toxin types (*2*–*4*). Toxin type E may also be produced by *C. butyricum* (*5*), and type F by *C. baratii* (*6*–*9*).

Botulism type F causes ≈1% of botulism cases in the United States (*10*). Two outbreaks have been reported, 1 in the United States, the other in Europe (*4*,*11*,*12*). A recent review described all 13 cases of botulism type F from the USA between 1981 and 2002 (*9*). Clinical signs were respiratory failure within 24 hours of symptom onset, complete or near complete quadriplegia by the fifth day, and neuromuscular recovery beginning on the eighth day. On average, patients received mechanical ventilation for 24 days and were hospitalized for 30 days. These features represent a more precipitous initial course than is typical for type A or type B botulism but a more rapid recovery. We describe a case of botulism type F in an adult who recovered despite the continued presence of toxin in the blood.

## The Case

In 2005, a 56-year-old woman sought treatment at the emergency department of a hospital in Helena, Montana, USA. She reported right-upper quadrant pain radiating to her back of 1 day’s duration and shortness of breath. Her medical history included hypertension, hyperlipidemia, and gastroesophageal reflux. The abdomen was tender at the right upper quadrant. Routine blood test results were unremarkable; an abdominal radiograph showed copious stool and gas. The patient had respiratory arrest in the emergency department and was intubated. She had unresponsive pupils dilated to 5 mm, minimal extraoccular muscle motion, facial paralysis, normal palatal and gag reflexes, near-paralysis of proximal upper and lower extremities but near normal muscle strength in the hands and feet, and symmetric deep tendon reflexes. Results of computed tomographic scans of the brain, chest, and abdomen were unremarkable. Cerebrospinal fluid values were within normal limits. An electromyogram (EMG) showed normal sensory nerve function, low amplitude on motor stimulation, mild (10%) decremental response on repetitive low frequency, and incremental response on high-frequency nerve stimulation, consistent with botulism (*13*).

Antitoxins to botulinum toxin types A, B, and E were administered within 24 hours. Nevertheless, paralysis progressed after antitoxin administration; within 48 hours, the patient was quadriplegic with no voluntary muscle function or distal tendon reflexes. On hospitalization day 4, a repeat EMG study showed no response to repetitive nerve stimulation.

The first improvement in neurologic status occurred on hospitalization day 8, when she moved her eyebrows, shook her head horizontally and laterally, lightly gripped, and plantarflexed and dorsiflexed her feet. Heart rate, which had been notably fixed at ≈70 beats/min, began varying for the first time. The Centers for Disease Control and Prevention (CDC; Atlanta, GA, USA) provided heptavalent (anti-ABCDEFG) equine F(ab′)2 antitoxin, but because of progressive clinical improvement, it was surmised that no toxin remained in circulation, and the antitoxin was not administered. Her neurologic function gradually improved; cranial nerve function was substantially improved by hospitalization day 12, although the pupils remained fixed for much longer. The first bowel movement, with enema, occurred on day 13. Distal tendon reflexes were first noted on day 17. On day 25, the patient combed her own hair. Weaning from mechanical ventilation was completed on day 35.

The patient underwent 1 week of in-hospital physical therapy, 3 hours per day. This period was marked by intermittent lightheadedness, orthostatic hypotension, and fatigue. Complications during hospitalization were aspiration pneumonia, *Candida glabrata* urinary tract infection with fungemia, heart failure attributed to diastolic dysfunction, and otitis media. She was discharged in stable condition on day 47. After hospitalization the patient reported that she required help getting out of bed and bathing for 1 month, help dressing for 2 months, and help getting up from a seated position for >7 months. She reported attaining pre-illness health 10 months after discharge.

Botulinum toxin type F was identified by mouse bioassay (*2*) at the Utah Public Health Laboratories in serum samples drawn on hospital days 1 and 8. Testing was confirmed at CDC. Test results were reported 21 days after serum collection, by which time treatment with specific antitoxin was not deemed necessary in view of substantial neurologic improvement. No protection was conferred on mice by antitoxins to botulinum toxins A, B, or E. A stool sample collected on hospital day 1 tested negative for botulinum toxin but yielded *C. baratii* that produced botulinum toxin type F. Public health officials did not identify any suspect food or other potential source of exposure.

## Conclusions

The clinical characteristics of this case closely resemble those in previously described adult botulism F patients (*9*). Our patient experienced respiratory collapse before her neurologic deficits were known, progressed to complete quadriplegia within 2 days, and showed the first signs of neurologic recovery on day 8. She was respirator dependent for 35 days and hospitalized for 45 days.

The patient had documented circulating botulinum toxin type F on hospital days 1 and 8. The assay’s limit of detection is ≈1 50% mouse intraperitoneal lethal dose (MIPLD_50_) per milliliter of patient blood. Therefore, estimating the patient’s circulatory volume at 5,000 mL whole blood and 3,000 mL plasma, this patient had a minimum of 3,000 MIPLD_50_ of type F toxin in circulation (apart from intracellular and bound toxin) on day 8 of hospitalization, the same day she demonstrated the first unequivocal signs of recovery from total quadriplegia. The type F toxin subcutaneous lethal dose in rhesus monkeys has been estimated at 25 MIPLD_50_/kg and the oral lethal dose at >4 × 10^6^ MIPLD_50_/kg (*3*). We cannot explain the patient’s clinical improvement in the face of circulating active toxin.

We were unable to determine whether the patient had foodborne or adult colonization botulism. Gupta et al. reported that, of 13 adult type F cases, 2 had confirmed adult colonization and 1 may have had foodborne botulism, but the syndrome in the remaining patients was not known (*9*). Infant type F botulism, by definition a colonization condition, does occur (*14*,*15*).

The economic burden of illness in this case was substantial. Apart from hospital charges of US $230,000, the patient required constant care by a relative and could not work for at least 7 months.

The similarity of the clinical features of this case with those previously described (*8*) indicate a highly predictable course of illness for botulism type F intoxication. Early suspicion of type F intoxication, suggested by specimens producing positive mouse assay results without protection of mice by injection of antitoxins for botulism toxins A, B, or E, may facilitate timely treatment with experimental type-specific antitoxin, available from CDC. Stool samples should be cultured over an extended period to assess for possible intestinal colonization.

We describe circulation of botulinum toxin in a patient on the day she demonstrated the first signs of recovery from complete quadriplegia. When future type F cases are identified by the presence of type F toxin in clinical specimens, the patient should be promptly treated with the appropriate antitoxin. The possibility of type F illness should be suspected if onset is rapid and paralysis is severe, and the laboratory should conduct immediate, specific testing. Intensive care support and antitoxin treatment are the standards for botulism care (*2*). Suspected cases of botulism of any type should be immediately reported to the state health department’s 24-hour emergency telephone number.
